# Endoscopic surgery to treat inverted papilloma: which are the limits?

**DOI:** 10.5935/1808-8694.20130050

**Published:** 2015-10-04

**Authors:** Erika Ferreira Gomes

**Affiliations:** a Specialist in General Surgery and Otorhinolaryngology (Graduate Student - Medical School of the University of São Paulo); Assistant Physician - General Hospital of Fortaleza SUS/SESA).

**Keywords:** natural orifice endoscopic surgery, nose neoplasms, papilloma, inverted

## INTRODUCTION

Endoscopic surgery has made substantial contributions to advance on the limits of nasal cavity, paranasal sinuses and skull base surgeries, with growing applications, especially in the treatment of tumors and reconstructive approaches. The paper published about a “Retrospective Analysis of 26 cases of nasal inverted papilloma”, showing the feasibility of the endoscopic approach to treat the inverted papilloma is very important.

## CASE PRESENTATION

In a review published about 26 patients (19.2%) there was tumor left over, there is no information whether the approach initially used in these cases was endoscopic, open or combined^1^.

## DISCUSSION

The recent publication shows that the inverted papilloma, when operated by endonasal endoscopy only, has a greater recurrence rate. In a series of 26 recurred tumors, 21 had been resected by the endoscopic approach^2^. The purely endonasal endoscopic approach provides for a broad access to the nasal cavity, medial wall and posterior wall of the maxillary sinus, frontal and sphenoid sinuses^3^. For tumors involving the anterior wall, the lateral or inferior wall of the maxillary sinus, it is useful to add a small sublabial incision for a combined transmaxillary access using the endoscope, which helps reduce the likelihood of residual tumor being left behind in these cases.

## FINAL REMARKS

External approaches, such as the Weber-Ferguson and the mid-facial degloving, have been replaced by the endoscope^4^. Despite all the technical advantages of the fully endonasal endoscopic approach, one must consider the combined transmaxillary technique - when operating T3 or T4 tumors - because of the high rate of recurrence inherent to the biological behavior of the tumor and the morbidity associated with reoperations in the affected age range^5^.
